# Primary Pulmonary Meningioma Simulating a Pulmonary Metastasis

**DOI:** 10.1155/2016/8248749

**Published:** 2016-11-16

**Authors:** Chun-Mao Juan, Mei-Ling Chen, Shang-Yun Ho, Yuan-Chun Huang

**Affiliations:** ^1^Department of Medical Imaging, Changhua Christian Hospital, No. 135 Nanxiao St., Changhua 500, Taiwan; ^2^Department of Pathology, Changhua Christian Hospital, No. 135 Nanxiao St., Changhua 500, Taiwan

## Abstract

Primary pulmonary meningiomas represent a rare tumor entity. Few cases have been reported in the English medical literature, and they have almost all been solitary and benign in nature, with the exception of several extremely rare cases. We report herein a case of PPM that raised suspicion of a pulmonary metastatic tumor initially, as it was depicted as a single, round, small, ground-glass opacity pulmonary nodule on a chest computed tomography scan, in a 55-year-old man with a history of buccal cancer. Increased awareness of the clinical and radiologic characteristics of this rare category can assist a multidisciplinary team to perform adequate management.

## 1. Introduction

Primary pulmonary meningiomas (PPMs) are rare tumors and only sporadic cases have been reported. Most PPMs are solitary pulmonary nodules, incidentally detected on chest plain film or chest computed tomography (CT). We report a case of PPM herein, which raised suspicion of a pulmonary metastatic tumor initially.

## 2. Case Report

**Figure 1:**
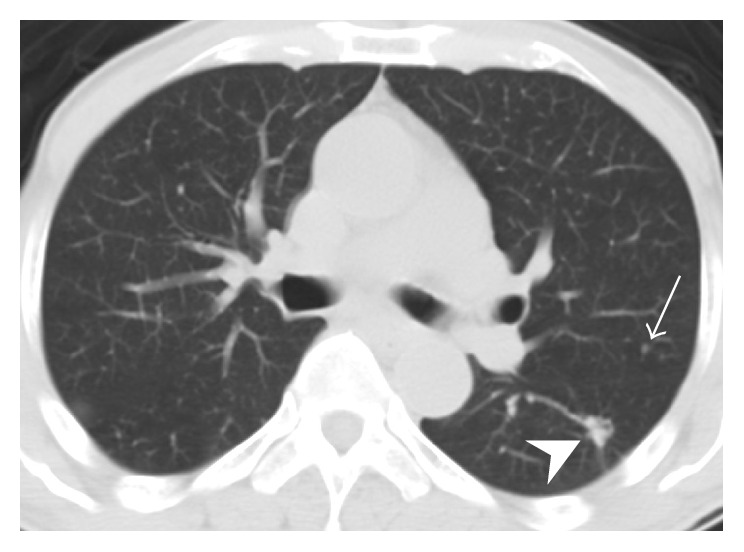
Axial chest computed tomography scan at subcarinal level demonstrates a 4.5 mm ground-glass opacity nodule in left upper lobe of lung (arrow) and a 16 mm part-solid ground-glass nodule in left lower lobe of lung (arrowhead). They are proved to be a pulmonary meningioma in left upper lobe of lung and granulomatous inflammation in left lower lobe of lung in histologic diagnosis, respectively.

A 55-year-old man was referred to our Chest Department due to abnormal pulmonary nodules located in the left upper and lower lung lobes, which were detected on a chest CT scan. He had a past history of left buccal cancer without distant metastasis more than ten years ago, for which he received surgery and local radiotherapy. He underwent regular follow-up by contrast-enhanced CT scanning of the head and neck without local recurrence. With the exception of buccal cancer, the patient denied any history of other neurologic or pulmonary disorders. He underwent a health examination, and a chest CT scan revealed a 4.5 mm ground-glass opacity nodule in the left upper lobe and a 16 mm part-solid ground-glass nodule in the left lower lobe of the lung (Figure 1). Three months later, the pulmonary lesions did not show regression in a follow-up chest CT. Due to a history of buccal cancer, the patient underwent thoracoscopic wedge resection of the pulmonary nodules owing to concern regarding pulmonary metastasis, with an uneventful recovery.

**Figure 2:**
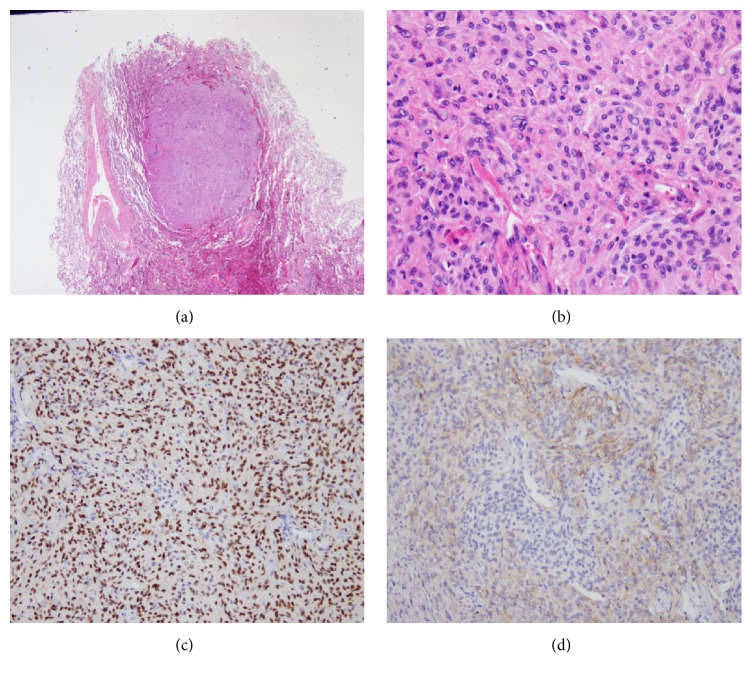
Histological features of the case (surgical lung biopsy specimen). (a) Microscopic examination revealed a well-defined nodule that compressed surrounding lung parenchyma. Hematoxylin-eosin (HE) 40x. (b) At high power view, the tumor includes nests of round to spindle shape cells that present focal whorls pattern. The tumor cells showed clear cytoplasm and round and oval nuclei with delicate chromatin distribution and some intranuclear inclusions. Mitotic figures are not present. Hematoxylin-eosin (HE) 200x. (c) In immunohistochemistry study, the tumor cells showed positive staining for PR (100x) and (d) CD56 (100x).

In histopathological study, the nodular lesion in the left lower lobe of the lung proved to be granulomatous inflammation. Microscopically, the tumor in the left upper lobe of the lung was composed of nests of round to spindle-shaped cells, which presented in a focal whorl pattern (Figure 2). The tumor cells showed clear cytoplasm and round and oval nuclei, with a delicate chromatin distribution and some intranuclear inclusions. They did not demonstrate mitotic figures nor atypia. Immunohistochemical staining demonstrated consistent expressions of epithelial membrane antigen (EMA), progesterone receptors (PR), and CD56 in the tumor cells. Conversely, the results of tests for S-100 protein, human melanin black 45 (HMB45), synaptophysin, and melan-A were negative. Finally, a histological diagnosis of pulmonary meningioma without characteristics of malignancy was made according to the above-described morphological and immunohistochemical features. Central nervous system surveys revealed negative findings. No tumor recurrence was observed in the 6-month follow-up CT study.

## 3. Discussion

PPM, defined by the typical histological features of meningioma in the absence of CNS lesions, is a rare neoplasm. Only 45 cases were described in the English medical literature to 2015 [1]. Most of these were solitary and benign in nature, and fewer than five cases have been reported to be malignant, with only one case of autopsy-proven liver metastasis [2–4]. Only one case was reported to consist of multiple primary pulmonary meningiomas. According to a review in 2008, this neoplasm appears to have a mild preponderance in older women [5]. The female/male ratio is 14 : 11, and the tumor size of the PPM ranges from 0.4 cm to 6.5 cm.

Most PPMs present as an asymptomatic solitary lung nodule and are detected incidentally on chest plain film or chest CT. Few cases have been reported to be symptomatic and present with hemoptysis [6]. On chest CT, a PPM usually presents as a single noncalcified pulmonary tumor with a circumscribed margin. Except in the case of endobronchial PPM, the bronchi or pleurae are not involved [7]. After contrast medium administration, the enhancement pattern is variable. Strong and homogeneous enhancement has been reported [1]. As in our case and in previously reported cases, when patients have a history of malignant tumor, PPM or isolated metastasis may not be distinguishable only on radiologic images. In a scenario of a pulmonary small nodular lesion which was detected in chest CT in a patient with history of malignancy like our patient, a metastatic tumor should be listed in the differential diagnosis. It was not distinguishable only on radiologic study if the lesion is small, because many tumors may present as a small round nodule initially. A tissue proof of the small pulmonary nodule with pathohistological study is needed if a definite diagnosis is required in this scenario [8].

Histologically, PPMs appear as well-circumscribed tumors without any bronchial or pleural involvement. Microscopically, these tumors usually present with spindle-shaped or ovoid cells arranged in lobules or a whorl pattern [9]. Absence of mitoses is most common in benign cases; conversely, mitoses are prominent in malignant cases. Psammoma bodies have often been reported. Immunohistochemistry demonstrates positivity for vimentin and EMA [10]. The incidence of extracranial meningioma metastasis is about 0.1%. The lungs (60%) are the most commonly involved locations of metastases of meningioma, followed by the abdomen (34%), cervical lymph nodes (18%), skeletal system (11%), pleura (9%), brain and spine (7%), and mediastinum (5%) [11]. Metastatic pulmonary meningioma is usually detected after diagnosis of central nervous system meningioma and may arise many years after excision of the primary meningioma. The interval from diagnosis of central nervous system meningioma to diagnosis of pulmonary metastases ranges between 2 months and 26 years [12]. Malignant histology results, a papillary morphology of tumor cells, the presence of venous sinus invasion, and local tumor recurrence are risk factors for distant metastasis. A histological diagnosis of pulmonary meningioma could represent a primary or secondary pulmonary meningioma, because PPM and metastatic meningioma share a similar histological appearance which presents as spindle-shaped or ovoid cells arranged in lobules or a whorl pattern [9, 13]. It is not possible to distinguish between primary and metastatic meningioma only by histologic study. Radiological study of the central nervous system is required to exclude an intracranial or spinal meningioma. Otherwise, magnetic resonance imaging is preferred to CT owing to its greater sensitivity [14].

Two hypotheses regarding the pathogenesis of PPMs have been proposed: they may arise from pluripotential subpleural mesenchyma or from minute pulmonary meningothelial-like nodules (MPMNs) [5]. Recently, some authors have performed studies that support the hypothesis that PPMs and MPMNs are related, because they may be derived from the same precursor cells [15]. MPMNs are small interstitial lesions (100 *μ*m to 3 mm) that maintain the original architecture of lung parenchyma, whereas PPMs present as nodular lesions that substitute for the parenchyma. MPMNs may have a relationship with chronic lung disease, whereas they may not be congenital cases due to absence in pediatric lung specimens [16]. Immunohistochemistry of MPMNs demonstrates positivity for CD56, progesterone receptor, epithelial membrane antigen, and vimentin, which is similar to PPMs. In fact, it may be difficult to distinguish MPMNs from PPMs, with the exception of clues by their size. Some authors have hypothesized that PPMs might be a giant form of MPMNs [17]; however, it is not known whether there is a great difference between the incidence of MPMNs (0.3 to 9.5% at autopsy or surgical resection) and that of PPMs.

The treatment for pulmonary meningioma is most commonly surgical resection. Wedge resection is ideal for peripheral lesions. On the contrary, lobectomy is suitable for lesions located in the central lung [5]. Recent widely accepted techniques such as CT-guided dye injection or coil placement could assist with localization of pulmonary nodules before wedge resection of small pulmonary nodules, guiding video-assisted thoracic surgery and minimizing sacrifice of pulmonary tissue to achieve an adequate diagnostic yield for histopathologic assessment. Surgical resection provides not only a conclusive pathologic diagnosis but an excellent long-term prognosis. No tumor recurrence has been reported in benign cases. Therefore, some authors consider that these tumors may be suitable for surgical resection due to their benign biological nature [18].

In conclusion, a greater knowledge of the clinical and radiological features of this rare entity is important to achieve more accurate and improved management. PPM should be listed in the differential diagnoses of single or multiple pulmonary nodules, even though it may present as a ground-glass opacity nodule on a CT scan.
